# VerSoX B07-B: a high-throughput XPS and ambient pressure NEXAFS beamline at Diamond Light Source

**DOI:** 10.1107/S1600577524001346

**Published:** 2024-03-26

**Authors:** David C. Grinter, Pilar Ferrer, Federica Venturini, Matthijs A. van Spronsen, Alexander I. Large, Santosh Kumar, Maximilian Jaugstetter, Alex Iordachescu, Andrew Watts, Sven L. M. Schroeder, Anna Kroner, Federico Grillo, Stephen M. Francis, Paul B. Webb, Matthew Hand, Andrew Walters, Michael Hillman, Georg Held

**Affiliations:** a Diamond Light Source, Diamond House, Didcot OX11 0DE, United Kingdom; b Lawrence Berkeley National Laboratory, Berkeley, CA 94720, USA; c University of Manchester, Manchester M19 3PL, United Kingdom; d School of Chemical and Process Engineering, University of Leeds, Leeds LS2 9JT, United Kingdom; e School of Chemistry, University of St Andrews, St Andrews KY16 9ST, United Kingdom; Advanced Photon Source, USA

**Keywords:** XPS, NEXAFS, ambient pressure, high throughput, X-ray photoelectron spectroscopy

## Abstract

The beamline optics and endstations at branch B of the Versatile Soft X-ray (VerSoX) beamline B07 at Diamond Light Source are described. B07-B provides medium-flux X-rays in the range 45–2200 eV from a bending magnet source, giving access to local electronic structure for atoms of all elements from Li to Y. It has an endstation for high-throughput X-ray photoelectron spectroscopy (XPS) and near-edge X-ray absorption fine-structure (NEXAFS) measurements under ultrahigh-vacuum (UHV) conditions. B07-B has a second endstation dedicated to NEXAFS at pressures from UHV to ambient pressure (1 atm). The combination of these endstations permits studies of a wide range of interfaces and materials. The beamline and endstation designs are discussed in detail, as well as their performance and the commissioning process.

## Abstract 2

This manuscript describes a new beamline for soft X-ray spectroscopy – VerSoX B07-B – at Diamond Light Source, UK. The beamline is designed to accommodate a wide range of sample environments and measurement conditions ranging from ultrahigh vacuum to *in situ* liquid studies.

## 1. Introduction

The Versatile Soft X-ray (VerSoX) beamline B07 at Diamond Light Source (DLS) has a very diverse user base across all branches of science and engineering, including studies of heterogeneous catalysts, batteries, biomaterials, sensors, functional energy materials, ionic liquids, protective coatings and pharmaceuticals. Soft X-ray photoelectron spectroscopy (XPS) and X-ray absorption spectroscopy (XAS), also termed near-edge X-ray absorption fine-structure (NEXAFS) spectroscopy, are ideal techniques to probe local interactions at the surfaces and in sub-surfaces of these materials. XPS and XAS provide elemental, chemical and structural information about solids, liquids and gases, as well as the interfaces between these phases (*e.g.* solid–gas, solid–liquid, liquid–vapour interfaces). The popularity of laboratory XPS as a standard surface characterization technique is well established (Greczynski & Hultman, 2020 ▸; Stickle & Young, 2019 ▸). Synchrotron-based XPS provides a number of additional advantages, especially a variable photon energy and a high flux of photons across the whole band of available photon energies. Variation of the photon energy can thus be utilized to vary the information depth of the spectra from bulk to very high surface sensitivity, increase photoabsorption cross-sections and facilitate resonant photoemission techniques.

Although its roots lie in ultra-high vacuum (UHV) studies of solids and their interactions with the gas phase (Chen, 1997 ▸; Tamenori, 2013 ▸), NEXAFS with soft X-rays has emerged as a versatile analytical tool for a broad range of applications, with near-ambient pressure operation facilitating even *operando* or *in situ* analysis of materials. Contemporary applications span both fundamental and applied research, including fields such as catalysis (Eren *et al.*, 2020 ▸; Liu *et al.*, 2023 ▸; Tolosana-Moranchel *et al.*, 2023 ▸), studies of liquids (Smith & Saykally, 2017 ▸), corrosion (Dwivedi *et al.*, 2017 ▸), biological materials (Akabayov *et al.*, 2005 ▸), batteries and electrolytes (Yang & Devereaux, 2018 ▸; Ye *et al.*, 2017 ▸; Swallow *et al.*, 2023*a* ▸), ionic liquids (Fogarty *et al.*, 2017 ▸; Seymour *et al.*, 2022 ▸), molecular orientation at surfaces (Dover *et al.*, 2020 ▸; Stoodley *et al.*, 2023 ▸), art conservation (Willneff *et al.*, 2014 ▸), local bonding of organic molecules in crystals (Edwards *et al.*, 2022 ▸), speciation in solution (Stevens *et al.*, 2015 ▸) and geochemistry (Koike *et al.*, 2020 ▸).

**Figure 1:**
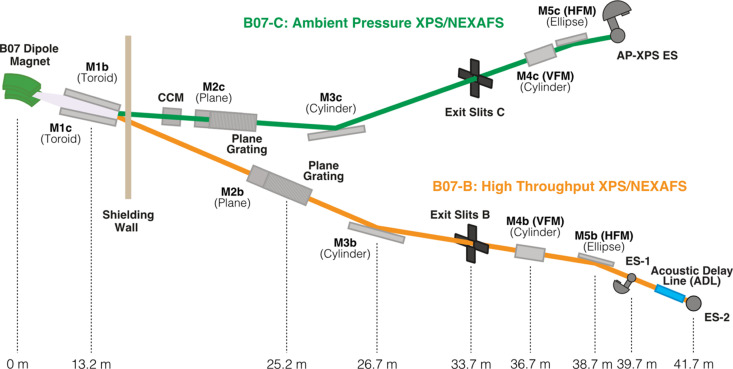
Schematic layout of the VerSox B07 beamline; distances indicated are measured from the dipole magnet source. Reproduced with permission from Grinter *et al.* (2022 ▸).

The VerSoX beamline B07 is split into two branches, B and C, each with dedicated optics to enable simultaneous, independent operation (Grinter *et al.*, 2022 ▸). The beamline layout for the two branches is shown schematically in Fig. 1 ▸. Branch C provides an ambient-pressure XPS facility that has been described in detail previously (Held *et al.*, 2020 ▸). The focus of this paper is on branch B, which provides soft X-rays for two endstations dedicated to high-throughput UHV XPS (ES-1) and ambient pressure NEXAFS (ES-2), respectively. The soft X-ray photon energy range between ∼50 eV and ∼2200 eV provided by B07-B includes key absorption edges, such as the *K*-edges from lithium to phospho­rus (atomic numbers *Z* = 3 to 15) and the *L*-edges from magnesium to yttrium (*Z* = 12 to 39), which include all first-row transition metals. The combination of fluorescence and electron yield detection techniques results in variable depth sensitivity for XAS measurements from a few nanometres for electron-yield (Schroeder, 1996 ▸) to over 100 nm for soft X-ray fluorescence, which cannot be achieved by XPS in this energy range.

## 2. Beamline B07-B

B07-B has two endstations which have been designed to accommodate studies under UHV as well as investigations of samples incompatible with UHV conditions or experiments that require high-pressure gas reactor cells. Combined, the two endstations cover the pressure range from 1 × 10^−10^ mbar to 1 × 10^3^ mbar. This extended pressure range complements the near-ambient-pressure XPS (NAP-XPS) branch C of the beamline, which has been in operation since 2017 (Held *et al.*, 2020 ▸; Grinter *et al.*, 2022 ▸).

### 2.1. Beamline design

The two branches of B07 share a similar overall design with the aim to provide a good photon energy resolution (*E*/Δ*E* > 5000) over a wide energy range (B: 45 eV to 2200 eV; C: 120 eV to 2800 eV) and with a photon flux >10^10^ photons s^−1^ (Held *et al.*, 2020 ▸; Grinter *et al.*, 2022 ▸). A dipole-bending magnet (1.4 T) serves as the source for both branches, emitting horizontally polarized radiation in a fan of ∼30 mrad horizontal width. A toroidal mirror (M1b) (optical surface 1380 mm × 20 mm; reflection angle 2.6°) captures 2.4 mrad of the bending magnet radiation fan, collimates the beam vertically and focuses it horizontally at the exit slit. Owing to the large size of the mirror, the mounting and clamping of internal water-cooling tubes caused distortions in its focal length and slope errors outside of the design parameters. This is compensated for by gentle counter heating (∼30 °C) using a dedicated closed-loop chiller (Hand *et al.*, 2019 ▸) and the use of clean up slits before the monochromator to discard the periphery of the beam which is worst-affected by the distortion. The pink beam enters a collimated plane grating monochromator (cPGM) manufactured by FMB Berlin, and the monochromatic beam is then focused vertically by a cylindrical mirror (M3b) onto the exit slits. The PGM is equipped with three gratings with line densities of 400 lines mm^−1^, 600 lines mm^−1^ and 1000 lines mm^−1^ to deliver the desired resolving power and flux across the energy range of the beamline. The choice of a cPGM permits the use of a variable fixed focus constant (*c*_ff_) condition, which can be utilized to provide absolute energy calibration, higher-order light suppression and higher resolution with a trade-off in flux, if desired (Follath, 2001 ▸; Weiss *et al.*, 2001 ▸). Two pairs of refocussing mirrors (M4b1/2 – vertical; M5b1/2 – horizontal) focus the beam onto the sample positions ES-1 and ES-2, respectively. Each mirror has independent switching and fine positioning mechanics. Polished copper plates facing the optical surfaces of the two M5b mirrors collect emitted photoelectrons and thus provide an *I*_0_ measurement for normalization of X-ray absorption spectra with respect to the beamline transmission. All of the beamline optics are maintained in an O_2_ partial pressure around 3 × 10^−8^ mbar to prevent carbon build up on the mirror and grating surfaces (Risterucci *et al.*, 2012 ▸; Held *et al.*, 2020 ▸). The layout and technical details of the main optical elements are summarized in Fig. 1 ▸, and Fig. S1 and Table S1 of the supporting information. The beamline is equipped with various diagnostic tools, including metal and fluorescent (YAG:Ce) screens for beam characterization, metal foils and meshes (Cu, Au) for relative beam intensity and *I*_0_ measurements, photodiodes for absolute flux measurement, quadrant beam position monitors (QBPMs) for auto-alignment of the switching mirrors, and a purpose-designed gas cell located downstream of the exit slits for characterizing the monochromator resolution.

### 2.2. Beamline performance

**Figure 2:**
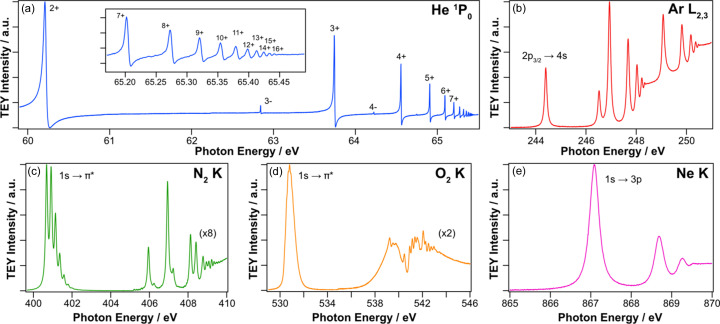
Gas phase TEY XAS spectra used to determine the beamline resolution. (*a*) He 1*s* ^2^ → (2*spn* ± 2*pns*) double excitation. (*b*) Ar *L*_2,3_-edge of Ar. (*c*) N *K*-edge of N_2_. (*d*) O *K*-edge of O_2_. (*e*) Ne *K*-edge of Ne. All spectra were obtained with the 600 l mm^−1^ grating and the smallest vertical exit slit opening of 4 µm.

Measurements of the energy resolution and calibration of the PGM are performed using the gas cell. This cell can be isolated from the vacuum of the rest of the beamline by inserting two 50 nm-thick aluminium membranes mounted on gate valves, thus allowing it to be pressurized up to ∼1 × 10^−1^ mbar. The photoionization current is measured from a thin copper wire running parallel to the beam path, which is at a potential of +36 V with respect to a coaxial steel cylinder of approximately 20 mm diameter. Using a series of inert gases with sharp X-ray absorption resonances, it is possible to determine the beamline resolution over a good portion of its energy range: He 1*s* ^2^ → (2*spn* ± 2*pns*) double excitation (62.84 eV), Ar *L*_2_ (244.4 eV), N *K* (400.8 eV) and Ne *K* (867.1 eV), as shown in Fig. 2 ▸.

There are three main contributions to the observed widths of the absorption features: the intrinsic lifetime broadening of the resonance, Γ, with a Lorentzian or Fano lineshape; a Gaussian lineshape dominated by the slope errors of the beamline optics; and the exit slit opening whose contribution is also dependent on the dispersion of the grating. For the rest of this work, we refer to the photon energy resolution (Δ*E*), which is combination of the Gaussian width and a box function representing the exit slit opening (Δ*E* ^2^ = FWHM_G_^2^ + FWHM_box_^2^).

**Figure 3:**
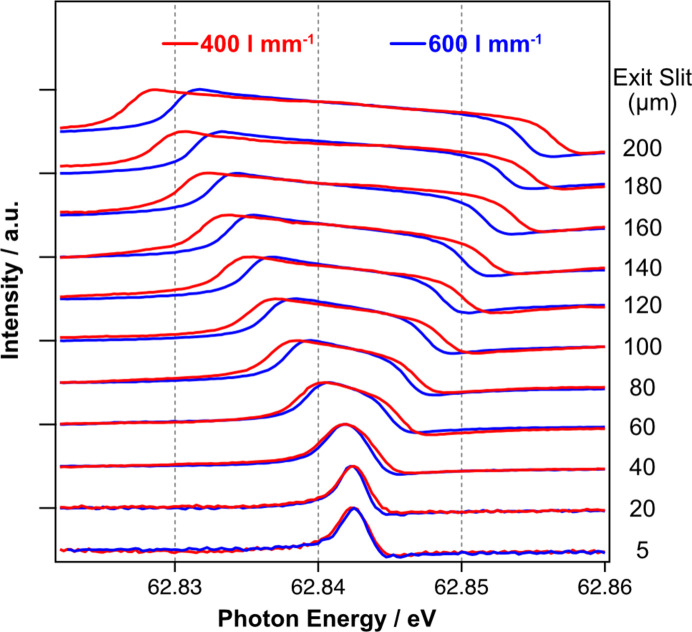
The 3- state of the helium 1*s* ^2^ → (2*spn* ± 2*pns*) double-excitation spectrum as a function of exit slit opening for the 400 l mm^−1^ (red) and 600 l mm^−1^ (blue) gratings. Spectra are offset vertically for clarity.

The photon energy resolution for the three gratings under optimal conditions (smallest exit slits of 4 µm) are summarized in Table 1 ▸. The helium 1*s* ^2^ → (2*spn* ± 2*pns*) double excitation spectrum provides an excellent test of the performance at low energies. Fig. 2 ▸(*a*) shows the series of very narrow Fano resonances in the range 60–66 eV. The inset shows the higher states where it is possible to resolve up to the 16+ level (Rost *et al.*, 1997 ▸). The 3- state has a very narrow Lorentzian width (0.12 meV) (Rost *et al.*, 1997 ▸) and, as a result, the overall width (∼2 meV) is dominated by the beamline, as shown in Fig. 3 ▸. The argon 2*p*_3/2_ → 4*s* transition at 244.4 eV [Fig. 2 ▸(*b*)] has a Lorentzian width fitted at 111 meV, yielding a beamline contribution in the range 11–20 meV. The frequently used *K*-edge of N_2_ gas is shown in Fig. 2 ▸(*c*) with the vibrational fine structure of the 1*s* → π* resonance requiring fitting with seven individual Voigt functions to extract the beamline resolution. A high-resolution plot of this is displayed in Fig. S2(*a*). Due to the relatively large Lorentzian width (112 meV) and overlap between the vibrational components, there is significant uncertainty in the resultant fitting, but the beamline generally exhibits an ultimate resolution between 20 meV and 40 meV depending on the grating used. As expected, there is a clear pressure dependence on the width of the absorption resonances as demonstrated in Fig. S2(*b*) (Connerade *et al.*, 1973 ▸). Fig. 2 ▸(*d*) shows the O *K*-edge of O_2_ where again vibrational splitting of the main 1*s* → π* resonance can be observed. Due to the uncertainties in fitting these to measure the resolution, we have not used them in this case. For higher photon energies, we have used the Ne *K*-edge as shown in Fig. 2 ▸(*e*), in particular the 1*s* → 3*p* transition, which yields a resolution in the range 75–119 meV, depending on the grating used.

The effect of opening the vertical exit slits is most clearly demonstrated in the double-excitation spectrum for He gas shown in Fig. 3 ▸. Here the ‘3-’ resonance is shown as a function of the vertical exit slit opening from 4–184 µm for the 400 l mm^−1^ (red) and 600 l mm^−1^ (blue) gratings. The exit slit opening was recalibrated after installation on the beamline using telescopic imaging. At smallest exit slit settings, we observe a broadened Fano lineshape which progressively becomes larger as the exit slit is opened. The variation in resolution between the gratings is clearly evident at the larger exit slit positions. By fitting with a box function to represent the exit slit width convoluted with a Fano lineshape, the predicted dispersion of the gratings is confirmed.

**Figure 4:**
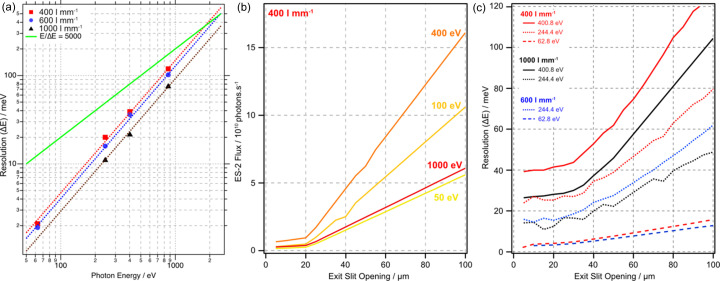
(*a*) Photon energy resolution (Δ*E*) as a function of photon energy for the three gratings, measured with a vertical exit slit opening of 4 µm (400 l mm^−1^: red squares; 600 l mm^−1^: blue circles; 1000 l mm^−1^: black triangles). (*b*) Photon flux at ES-2 from the 400 l mm^−1^ grating as a function of vertical exit slit opening for four photon energies. (*c*) Photon energy resolution (Δ*E*) as a function of vertical exit slit opening for the three gratings at 62.8 eV (He, dashed line), 244.4 eV (Ar, dotted line) and 400.8 eV. (N_2_, solid line).

The ultimate performance at the minimum exit slit opening of 4 µm is summarized in Fig. 4 ▸(*a*), where the photon energy resolution, as measured at the absorption edges for He, Ar, N_2_ and Ne, is plotted as a function of photon energy for each of the three gratings of the PGM. The fitted lines in Fig. 4 ▸(*a*) clearly show that the energy resolution follows the expected *E* ^3/2^ dependency (Follath, 2001 ▸). The green line represents a fixed resolving power of *E*/Δ*E* = 5000, the original design specification of the beamline. By extrapolation, the beamline exceeds this specification by a large margin for all gratings at energies <1000 eV and is expected to meet the specification up to the maximum photon energy of 2200 eV with the 600 l mm^−1^ and 1000 l mm^−1^ gratings.

As demonstrated in Fig. 3 ▸, opening the vertical exit slits increases the observed width of the absorption features, but there is a concomitant increase in photon flux at the sample, as shown in Fig. 4 ▸(*b*). These data show the flux off the 400 l mm^−1^ grating as measured with a photodiode at the sample position of ES-2 as a function of exit slit opening from 4 µm to 84 µm at four photon energies (50 eV: yellow; 100 eV: amber; 400 eV: orange; 1000 eV: red). All three show similar behaviour where there is the predicted linear increase with the exit slit opening. Fig. 4 ▸(*c*) shows the effect of opening the exit slits on the total beamline resolution for the three gratings (400 l mm^−1^: red; 600 l mm^−1^: blue; 1000 l mm^−1^: black) measured at three photon energies (62.8 eV: He; 244.4 eV: Ar; 400.8 eV: N_2_). There is a linear relationship between the resolution and gap at large exit slit openings; however, for values less than ∼30 µm the gain in resolution becomes progressively lower as would be expected given the size of the dipole source and beamline optical arrangement. Another key factor in the ultimate photon energy resolution is the slope error of M1b, both intrinsic and that induced by the distortion caused by the rigid copper cooling lines (Hand *et al.*, 2019 ▸). Fig. S3 demonstrates the effects of the counter heating (∼30 °C) we employ to correct the horizontal focus; changes on the order of degrees Celcius lead to a change in focal distance of ∼50 mm, and a change in horizontal beam width at the exit slits of up to 100%.

**Figure 5:**
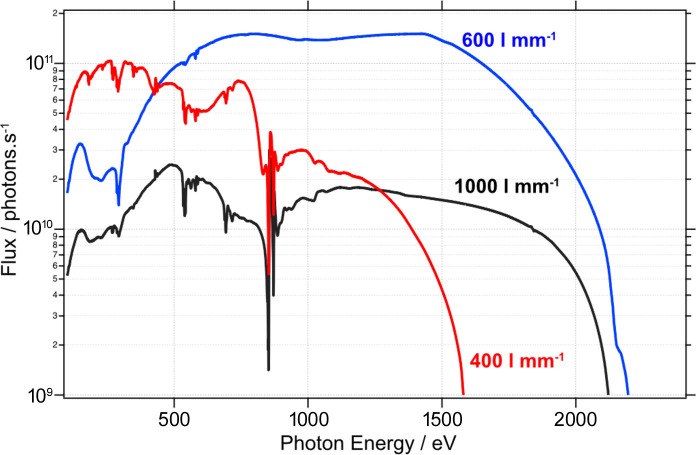
Beamline transmission for the three gratings as measured from a photodiode in ES-2, vertical exit slit opening 9 µm, *c*_ff_ 1.4.

The transmission of the beamline was measured using an AXUV-100G photodiode mounted at the sample position in ES-2 and is shown for the three gratings in Fig. 5 ▸. The 400 l mm^−1^ grating (red) is intended mainly for use at low energies <500 eV, the 600 l mm^−1^ (blue) provides good overall performance above 500 eV, and the 1000 l mm^−1^ (black) is intended for high-resolution measurements at high photon energies or for radiation-sensitive samples where a lower flux density might be desired. The 400 l mm^−1^ and 1000 l mm^−1^ gratings both have nickel coatings which give rise to significant absorption features at 850–870 eV (Ni *L*_2,3_-edge) and in the O *K*-edge region (from NiO) along with associated higher harmonics of these features, which require careful *I*_0_ correction during absorption measurements, although these gratings are not typically used to scan Ni edges. The high-energy cut-off of the beamline is controlled to some extent by the *c*_ff_ of the PGM and ultimately absorption at the Au *M*_4,5_-edge due to the coating of M1b. The photon flux above the Au *M*_4,5_-edge is approximately two orders lower; however, NEXAFS measurements of the S *K*-edge (2450 eV) are still possible in certain cases on samples with sufficiently high atomic concentration. A powerful feature of the collimated PGM design is the ability to vary *c*_ff_ to minimize the higher-order transmission (Follath, 2001 ▸; Weiss *et al.*, 2001 ▸), which B07-B is particularly susceptible to because of the high cut-off energy of M1b. By reducing *c*_ff_ from 2.25 to 1.40 (Fig. 5 ▸ displays the flux at *c*_ff_ 1.4), the second- (and higher-) order intensity is reduced by 95% (see the supporting information, Fig. S4) at the expense of a decrease in the resolving power by a factor of 2–3.

The beam size at the sample position of the two endstations was measured using knife-edge scans and by visualizing the beam spot on a fluorescent screen (YAG:Ce) as displayed in Fig. S5. In the horizontal direction, the beam is 150 (±20) µm-wide at ES-1 (normal incidence, 280 µm when rotated by 60° for normal emission XPS, see below) and 240 (±20) µm at ES-2. The height of the beam is defined by the vertical gap of the exit slits; at 30 µm, the largest opening normally used, it is 80 (±10) µm at ES-1 and 100 (±10) µm at ES-2. To avoid beam damage, samples in ES-1 can also be illuminated with the beam focused on ES-2, either horizontally, vertically or in both directions, thus spreading the same photon flux over a larger area and decreasing the flux density. The resultant beam sizes and flux density for a photon energy of 1200 eV and a resolution of 300 meV at normal emission geometry for XPS are shown in Table 2 ▸. The horizontal size of the beam can be further adjusted by a set of slits located between M1b and the PGM (see Fig. 1 ▸) to give an optimum match to the sample dimensions, if required.

## 3. Endstation 1: high-throughput XPS

**Figure 6:**
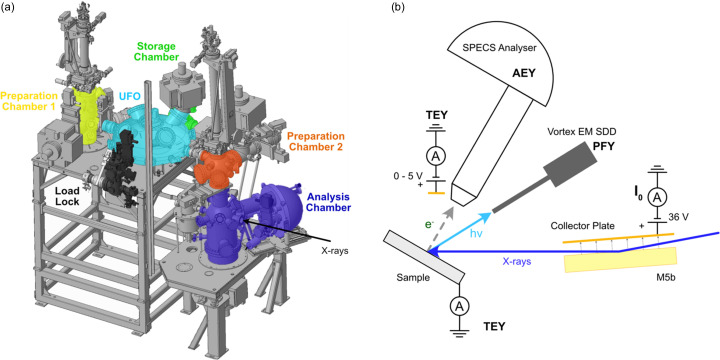
(*a*) 3D model of ES-1. Preparation chamber 1: yellow; load lock: black; rotary distribution chamber (UFO): light blue; storage chamber: green; preparation chamber 2: orange; analysis chamber: purple. Adapted with permission from Grinter *et al.* (2022 ▸). (*b*) Schematic of the various measurement techniques available in ES-1.

Endstation 1 (ES-1) is dedicated to UHV XPS and NEXAFS experiments with the option of fully automated in-vacuum sample motion and manipulation for high-throughput measurements. A labelled schematic model of the endstation is shown in Fig. 6 ▸(*a*) with a photograph shown in Fig. S6. Samples are introduced into a fast-entry load lock either from air or via a vacuum-suitcase which is also compatible with the B07-C NAP-XPS system. They are then distributed via a fully motorized and software-controlled rotary distribution chamber (‘UFO’) to either the main preparation chamber (preparation chamber 1), the sample storage chamber with space for six sample holders or a small preparation chamber above the analysis chamber (preparation chamber 2). The preparation chambers include equipment for standard UHV sample treatment and preliminary characterization, including an inert gas-sputter source, e-beam evaporator, residual gas analyser, leak valves for gas dosing and low-energy electron diffraction (LEED) optics. Preparation chamber 1 is equipped with a four-axis manipulator (*X*, *Y*, *Z*, polar rotation) and permits heating/cooling between 150 K and 1300 K. The manipulator in preparation chamber 2 also serves the analysis chamber and has five axes (*X*, *Y*, *Z*, polar and azimuthal rotations) and again offers heating/cooling between 150 K and 1300 K, dependent on the sample holder used. Gases, including toxic substances and high vapour pressure liquids, are delivered via a combination of manual and piezo-activated high-precision leak valves. A tungsten beam shutter (FMB Oxford) is located downstream of the analysis chamber, prior to the acoustic delay line, to allow maintenance of ES-2 while synchrotron experiments are performed in ES-1.

**Figure 7:**
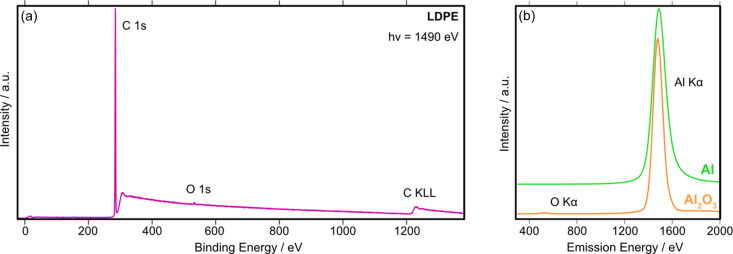
(*a*) XPS survey spectrum of LDPE, measured using a flood gun (60 V, 60 µA emission current). (*b*) X-ray emission spectrum (*h*ν = 1560 eV) of Al foil (green curve) and Al_2_O_3_ powder (orange curve).

Fig. 6 ▸(*b*) shows a schematic of the various measurement techniques available within ES-1. The analysis chamber is equipped with a hemispherical electron energy analyser (SPECS Phoibos 150) with a 2D-CMOS detector optimized for high transmission. The analyser is mounted with its axis horizontal to the floor at 60° relative to the incoming beam direction. Analyser control and data acquisition are fully integrated into the beamline control system (*EPICS*) and data acquisition software (*GDA*). This way the analyser data acquisition can be synchronized with the monochromator photon energy to record Auger electron yield (AEY) NEXAFS or resonant XPS scans, either by recording ‘snapshot’ or fixed transmission spectra. The analyser has a ‘small area’ lens mode which matches the beam size when the beam is focused on ES-1, and a ‘large area’ lens mode which matches the beam when the beam is focused on ES-2. A copper electrode is located facing the sample and is held at a small positive bias (5 V) and connected to a Stanford Research Systems SR570 current amplifier to provide the total electron yield (TEY) during NEXAFS measurements. A low-energy electron flood gun (SPECS) allows compensatory charging of insulating samples during photoemission measurements. Fig. 7 ▸(*a*) shows an XPS survey spectrum collected from a 1 mm-thick low-density polyethyl­ene (LDPE) foil where the sample charging is completely compensated by the flood gun. With the flood gun switched off, no photoemission peaks are visible at all.

Partial fluorescence yield (PFY) NEXAFS is provided by a Vortex EM silicon drift detector (Hitachi) with Xspress Mini 3 (Quantum Detectors) readout electronics. The detector is mounted on a retractable linear stage to minimized saturation/photon pile-up events from concentrated samples. Fig. 7 ▸(*b*) shows typical X-ray emission spectra from an aluminium foil (green curve) and Al_2_O_3_ powder (orange curve) obtained on resonance at the Al *K*-edge (*h*ν = 1560 eV). The energy resolution of the Vortex detector was measured using the Cu *L*α emission line (930 eV) from a clean copper foil at 89 (±5) eV (FWHM).

ES-1 uses the Prevac PTS sample holder system which is also used in the B07-C NAP-XPS endstation (see photograph in Fig. S7). The sample holders contain integrated heaters (options including resistive or electron bombardment), electrical contacts for thermocouples which can be directly attached to the samples to ensure accurate temperature measurement and additional sample bias connections. Quartz crystal microbalance holders and flag style sample plate (as used on ES-2) adapters are also available. A larger 50 mm × 50 mm sample plate is also available for mounting multiple samples for high-throughput measurements which do not require heating (see photograph in Fig. S7).

A dedicated software perspective has been developed within *GDA* to handle these large sample plates where up to 100 samples could be mounted at a time. A high-resolution photograph of the plate is taken in the sample-handing laboratory prior to introduction into the endstation, and users can register and label the samples as well as choosing precise locations on them to perform XPS and/or NEXAFS scans. The software then automatically performs these scans, moving the plate to the desired locations without requiring further user input, enabling easier remote operation of the beamline and increasing throughput. Precise, rapid calibration of the photon energy during XPS experiments is possible by exploiting the presence of second-order X-rays as described in Fig. S8. Since commissioning was completed in 2022, the endstation has been used for a variety of user experiments including high-throughput measurements and traditional single-crystal surface science studies (Tolosana-Moranchel *et al.*, 2023 ▸; Stoodley *et al.*, 2023 ▸).

## 4. Endstation 2: ambient-pressure NEXAFS

**Figure 8:**
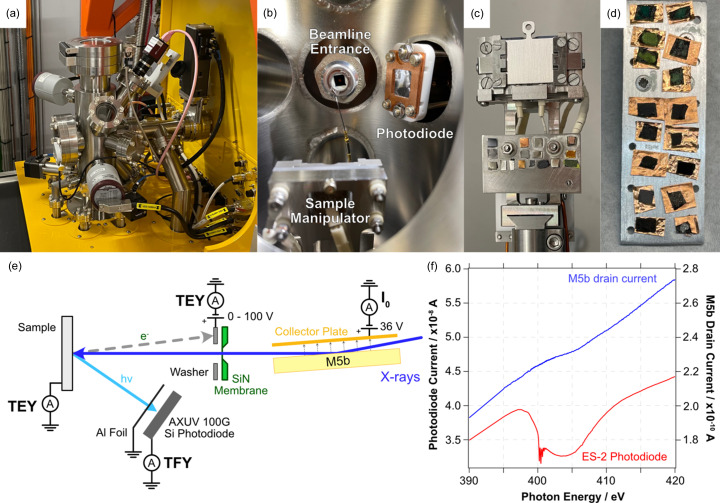
(*a*) Photograph of the ES-2 vacuum chamber. (*b*) Photograph inside ES-2 highlighting the sample manipulator, beamline entrance and photodiode. (*c*) ES-2 sample manipulator. (*d*) ES-2 multi-sample plate. (*e*) Measurement principle schematic. (*f*) Comparison of the *I*_0_ correction provided by the M5b drain current (blue curve) and the photon flux in ES-2 (red curve) across the N *K*-edge.

Endstation 2 (ES-2) is located 2.0 m downstream of ES-1; the two are separated by an acoustic delay line (ADL) chamber as shown in the 3D model in Fig. S9. ES-2 is designed for NEXAFS measurements of solid, liquid and gaseous samples with a pressure range from 10^−7^ mbar to 1 bar. A photograph of the vacuum chamber is shown in Fig. 8 ▸(*a*). Samples are introduced via a fast entry door on the top of the chamber, and mounted either directly on flag-style sample plates [Fig. 8 ▸(*c*)] or on custom-made multi-sample holders [Fig. 8 ▸(*d*)] if heating is not required, which fit onto the four-axis sample manipulator [Fig. 8 ▸(*b*)]. This manipulator is capable of heating to 400 °C under UHV or around 200 °C under 1 bar, depending on the gas. The lower part of the sample holder contains a number of standard samples for energy referencing [Fig. 8 ▸(*c*)]. The high-pressure environment of the endstation is separated from the UHV of the beamline by a 75 nm-thick silicon nitride (SiN_*x*_) membrane, which is glued to a quick-torr fitting for rapid exchange in case of breakage [Fig. 8 ▸(*b*)]. If this membrane fails, the ADL delays the inrush of gas (Betz *et al.*, 1979 ▸) for long enough (>10 ms) to enable a fast-closing valve (VAT 75 series) to shut, thus protecting the vacuum of ES-1 and the beamline optics. The entire endstation is enclosed in a small radiation hutch, which ensures user safety irrespective of modifications to the internal setup, which might cause radiation leaks.

Inert and non-toxic gases are supplied to the endstation via manual leak valves and via an automated gas inlet system for standard measurements under 1 mbar He. To enable transfer of samples under inert conditions, a vacuum suitcase has been developed that is compatible with most commercial glove boxes, and can be adapted to suit other vacuum systems that use similar flag style sample plates. The measurement principles for NEXAFS experiments in ES-2 are shown in the diagram in Fig. 8 ▸(*e*). Total electron yield (TEY) is measured either via the sample drain current or off a positively biased (108 V) electrode glued to the SiN_*x*_ membrane of the beamline entrance [Fig. 8 ▸(*b*)] using a Stanford Research Systems SR570 low-noise current amplifier. This latter method is particularly suited for the measurement of insulating samples. For total fluorescence yield (TFY) measurements, a photodiode (AXUV-100 G TF400) is used which has a 150 nm aluminium coating to block photoelectrons and stray visible light [Fig. 8 ▸(*b*)]. A future upgrade of the ES-2 chamber will permit mounting of the Vortex SDD PFY detector, although the fragility of its entrance window must be considered when using high-pressure gas or liquid cells. To correct for structure in the beamline transmission and provide *I*_0_, the photocurrent off the final refocussing mirror (M5b) is measured simultaneously with any NEXAFS data, using a biased collector plate [Fig. 8 ▸(*e*)]. When measuring the N *K*-edge or Si *K*- or *L*-edges of samples containing low atomic concentrations of these elements, it is important to accurately correct for any absorption by the silicon nitride window. This is most easily achieved by backfilling the chamber with a few millibar of helium and monitoring the current on the beamline entrance electrode over the absorption edge of interest. In Fig. 8 ▸(*f*), the energy range of the N *K*-edge is shown with only minor variations in the M5b mirror drain current (blue line), whereas the ES-2 signal (red line) shows a clear dip from the silicon nitride, along with the characteristic features at 400.8 eV from trace N_2_ impurities in the He gas. Energy calibration of soft X-ray NEXAFS is typically via comparison with known standard samples from the literature. At the C and O *K*-edges, characteristic features in the beamline transmission (acquired simultaneously by monitoring the M5b current) also allow quick energy calibration (see Fig. S10). UV illumination is provided at the sample position by coupling of a 365 nm LED UV source via in-vacuum fibre-optics.

**Figure 9:**
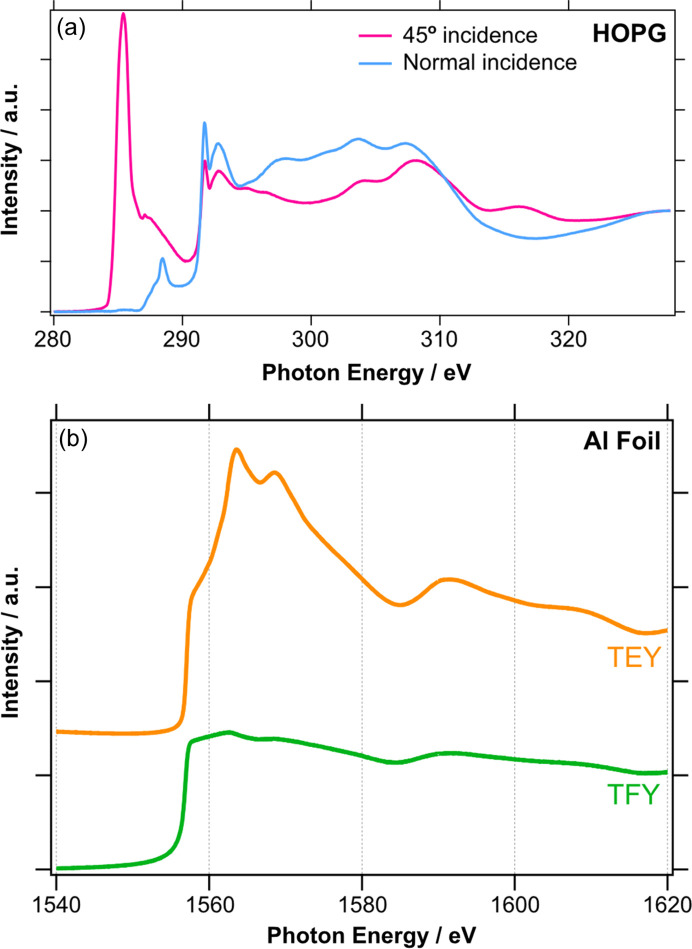
(*a*) C *K*-edge NEXAFS of HOPG at normal incidence (blue) and 45° incidence (pink). (*b*) Comparison of TEY (orange) and TFY (green) at the Al *K*-edge of an Al foil.

Fig. 9 ▸(*a*) shows C *K*-edge TEY NEXAFS spectra of highly oriented pyrolytic graphite (HOPG) at normal and 45° incidence. The 1*s* → π* transition at 285.4 eV is symmetry-forbidden at normal incidence when the polarization vector is parallel to the planes of the carbon atoms and therefore not observed in the corresponding spectrum (blue). This sensitivity of NEXAFS to the surface anisotropy can be used to quantify the degree of beamline polarization as described by Watts *et al.* (2006 ▸). Following this methodology, we measure the degree of linear polarization under standard beamline operating conditions of 92 ± 4% (Fig. S11). Fig. 9 ▸(*b*) compares the TEY (orange) and TFY (green) NEXAFS spectra of the Al *K*-edge of aluminium foil, which was exposed to air. The TFY signal is that of bulk Al, whereas the more surface sensitive TEY signal also has features due to the native oxide layer at the surface.

NEXAFS measurements at the *K*-edge of low atomic number elements, such as those shown in Fig. 9 ▸, or *L*-edge of the first-row transition metals typically take 5–10 min to acquire in TEY mode for concentrated samples. Using integration times of typically 0.1 s per data point, the scan rate is limited by the movement of the PGM grating and mirror which are currently operated in a step-scan mode. A continuous scanning mode will be implemented as a future upgrade to increase the data acquisition rate and improve the overall throughput. To facilitate remote operation of the beamline, a dedicated *GDA* perspective was developed. This collects the most important beamline controls and live camera views into one window, along with live data-plotting and Python scripting to enable operation of ES-2 from a single computer display. A screenshot of this perspective is shown in Fig. S12. The typical mode of operation involves mounting up to 20 samples on a large sample plate [as shown in Fig. 8 ▸(*d*)] followed by the user defining measurement locations (sample manipulator coordinates) and desired NEXAFS scans in a Python script, which can be run automatically. Such scripts can control common experimental parameters such as temperature, gas dosing and beamline setup (gratings, slits *etc.*), and future improvements will also enable automatic setting of the amplifier sensitivities. This enables the beamline to deliver 24 h operation with minimal user presence required on-site.

## 5. Cells

ES-2 is particularly well suited for easy attachment of small cells to contain liquids or high-pressure gases for *operando* NEXAFS investigations, a popular approach for similar soft X-ray beamlines around the world (Castán-Guerrero *et al.*, 2018 ▸; Velasco-Velez *et al.*, 2014 ▸; Swallow *et al.*, 2023*b* ▸). Currently, two cells are available to users and operated on a regular basis: a micro-reactor for catalytic gas-phase reactions at pressures in the bar range and an electrochemical cell. Both are sealed with SiN_*x*_ membranes and can be operated in either TEY or TFY mode. More details can be found in the subsections below. In addition, mounts have been machined to hold ‘coin’ and other similar cells for *in situ* studies of batteries, also sealed with X-ray transparent SiN_x_ membranes and utilizing TFY detection.

### 5.1. Microreactor for *in situ* NEXAFS

**Figure 10:**
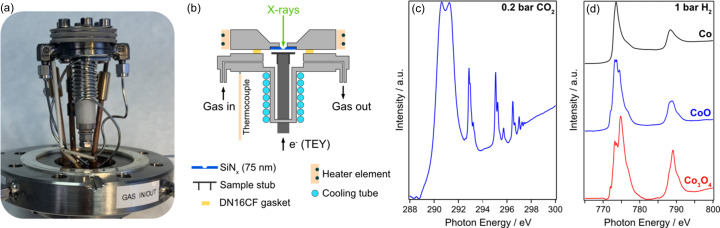
(*a*) Photograph of the B07-B ES-2 microreactor. (*b*) Schematic of the microreactor. (*c*) C *K*-edge TEY NEXAFS spectrum of 0.2 bar CO_2_ inside the microreactor. (*d*) Co *L*_2,3_-edge TEY NEXAFS of a cobalt oxide catalyst undergoing reduction in 1 bar of H_2_ at 300 °C.

A low-cost, reliable microreactor was developed in collaboration with the University of St Andrews, UK, with the aim of closing the pressure gap for the study of heterogeneous catalysts that require pressures in the 1 bar range. Figs. 10 ▸(*a*) and 10 ▸(*b*) show a photograph of the reactor and a schematic of the key components, respectively.

The microreactor has a volume of 400 µl; it is based on a standard DN16CF electrical feedthrough, to which a replaceable sample stub is attached [see Fig. 10 ▸(*b*)]. Catalyst samples are drop-cast onto the stub, which is connected to a current amplifier to record the TEY signal during NEXAFS measurements. The top of the reactor is capped by a DN16CF flange (standard Cu gasket) which has a hole machined and an SiN_*x*_ membrane (typically 100 nm thick) glued to the back of it to provide a path for the X-rays. The reactor is heated by a heating wire wrapped around insulating ceramics, through which up to 15 A current can be passed, allowing a maximum temperature of 450 °C (at 1 bar, measured by a K-type thermocouple attached to the body of the cell). The temperature at the sample position is between 20 °C and 40 °C lower than the indicated temperature of the thermocouple; look-up tables are used to precisely record the real temperature. To speed up cooling after high-temperature measurements, water can be passed through tubes at the back section of the reactor.

Gases, including toxic or flammable gases, such as CO or H_2_, are fed into the microreactor via 1/16′′ metal tubes from a mobile gas rig developed at DLS, with software-controlled mass-flow-controllers (MFCs) for up to eight gases, which are fully embedded into the beamline’s *EPICS* control software. In addition, mass spectrometry and gas chromatography are available to assess the reaction products *in situ*. Fig. 10 ▸(*c*) shows the C *K*-edge NEXAFS spectrum of 0.2 bar CO_2_. Fig. 10 ▸(*d*) shows *in situ* cobalt *L*_2,3_-edge NEXAFS spectra from cobalt oxide nanoparticles supported on alumina, as they are reduced in 1 bar H_2_ at 300 °C. The spectra in Fig. 10 ▸(*d*) show the clear progression from the prepared Co_3_O_4_ (red) to the active metallic Co catalyst, (black) via an intermediate CoO phase (blue).

### 5.2. Electrochemical cell

**Figure 11:**
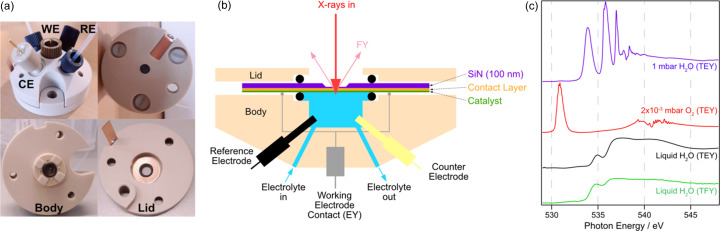
B07 *in situ* electrochemical cell. (*a*) Photographs of the assembled cell and the main components. (*b*) Schematic of the cell. (*c*) O *K*-edge NEXAFS of 1 mbar H_2_O (blue line), 2 × 10^−3^ mbar O_2_ (red line) and liquid water (TEY: black line; TFY: green line).

The electrochemical cell was developed in collaboration with Redox.me for *operando* X-ray spectroscopy of liquids and electro(photo)catalysts. The body of the cell, shown in Fig. 11 ▸(*a*), is machined from PEEK; the SiN_*x*_ membrane is held in place by O-rings of different materials dependent on the electrolyte. The membranes used typically have a 1 mm × 1 mm SiN_*x*_ window of 50–100 nm thickness on a 7.5 mm × 7.5 mm Si frame, which is 380 µm thick. A schematic of the main components of the cell is shown in Fig. 11 ▸(*b*). The working electrode consists of an SiN_*x*_ membrane coated with a conductive contact layer, typically 10 nm Ti/Au, on which the catalyst of interest is deposited. Standard Pt counter and Ag/AgCl micro-reference electrodes are also present. The cell can be operated either in two-electrode or three-electrode configuration, in static mode or with a constant flow of liquid via a syringe or peristaltic pump. Typical flow rates for the former are 600 µl min^−1^ to minimize the potential for damage to the delicate silicon nitride membranes. An industry standard potentiostat (Ivium CompactStat) can be used to control the electrode potential during *in situ* electrochemical measurements. Alternatively, the potential of a given electrode can be controlled in the range ±5 V by the in-built biasing capability of the Stanford SR570 current amplifier that is used for TEY detection.

The total volume of the cell is approximately 300 µl; liquid is supplied by 1/16′′ peek tubes, which are interlocked to the ES-2 pressure gauges to cut the flow in the event of the SiN_*x*_ membrane failing. The cell can be operated in TFY mode using the existing photodiode in the ES-2 chamber, or in TEY mode by connecting the working electrode to a current amplifier and grounding the counter electrode. As a future upgrade, there are plans to install a beam chopper to modulate the incoming X-rays and therefore the induced photo-induced current. This will enable detection of the TEY signal (typically picoamps) via a lock-in amplifier, in the presence of the much higher electrochemical current (typically milliamps) (Velasco-Velez *et al.*, 2014 ▸). Fig. 11 ▸(*c*) shows TEY NEXAFS spectra of the O *K*-edge of water vapour (1 mbar, blue curve) and O_2_ (2 × 10^–3^ mbar, red curve) as well as both TEY (black curve) and TFY (green curve) NEXAFS of liquid water, obtained from the cell. Some saturation effects are visible in the TFY measurement, typified by the suppression of intensity/flattening off in the region 536–542 eV.

## 6. Summary and conclusions

VerSoX B07-B at Diamond Light Source is a new beamline for soft X-ray spectroscopy across a broad photon energy range (45–2200 eV), covering sample environments from UHV to gas pressures in the 1 bar range and permitting studies of liquids. The beamline provides medium-flux X-rays from a bending magnet (10^10^ to 10^11^ photons s^−1^) with high energy resolution (up to *E*/Δ*E* = 30 000), focused on two endstations with spot sizes of 150 µm × 80 µm and 240 µm × 100 µm, respectively. ES-1 provides a UHV system for XPS and NEXAFS (TEY, AEY, TFY, PFY) with high-throughput measurement capabilities with motorized sample manipulation and control, and standard surface science preparation facilities. ES-2 provides a facility for rapid NEXAFS measurements under ambient conditions (10^−7^ mbar to 1 bar) as well as specialized sample environments including a microreactor for heterogeneous catalysis and an *operando* electrochemical cell. Advanced software control of the beamline and endstations maximizes sample throughput and ease-of-use, broadening the potential user base.

## Supplementary Material

Supporting information file. DOI: 10.1107/S1600577524001346/vy5019sup1.pdf

## Figures and Tables

**Table 1:** B07-B PGM ultimate photon energy resolution (vertical exit slit opening 4 µm) The lower energy bound of the 1000 l mm^−1^ grating is ∼100 eV.

			400 l mm^−1^		600 l mm^−1^		1000 l mm^−1^	
Gas	*E*_0_ (eV)	Γ (meV)	Δ*E* (meV)	*E*/Δ*E*	Δ*E* (meV)	*E*/Δ*E*	Δ*E* (meV)	*E*/Δ*E*
He	62.84†	0.12†	2.1	29900	1.9	33000	–	–
Ar	244.4‡	111‡	20	12000	16	15000	11	22000
N_2_	400.8‡	112	39	10000	36	11000	21.4	19000
Ne	867.1‡	250‡	119	7300	102	8500	75	12000

† Rost *et al.* (1997 ▸).

‡ Prince *et al.* (1999 ▸).

**Table 2:** Beam sizes at ES-1, measured at normal emission geometry for XPS, at different settings of the M4b/M5b refocusing mirrors The flux density is calculated for a photon energy of 1200 eV at a resolution of 300 meV.

M4b focus	M5b focus	Vertical beam size (µm)	Horizontal beam size (µm)	Flux density (photons s^−1^ µm^−2^)
1	1	80	280	1.3 × 10^7^
1	2	80	3200	1.2 × 10^6^
2	1	920	280	1.2 × 10^6^
2	2	920	3200	1.0 × 10^5^
