# Supplementary material for: VerSoX B07-B: a high-throughput XPS and ambient pressure NEXAFS beamline at Diamond Light Source
Source: J Synchrotron Radiat. 2024 Mar 26;31(Pt 3):578–89. doi: 10.1107/S1600577524001346 (PMC11075707; doi:10.1107/S1600577524001346)
Supplement: Supplementary file 1 [file s-31-00578-sup1.pdf]

JOURNAL OF SYNCHROTRON RADIATION

Volume 31 (2024)

Supporting information for article:

VerSoX B07-B: a high-throughput XPS and ambient pressure NEXAFS beamline at Diamond Light Source

David C. Grinter, Pilar Ferrer, Federica Venturini, Matthijs A. van Spronsen, Alexander I. Large, Santosh Kumar, Maximilian Jaugstetter, Alex Iordachescu, Andrew Watts, Sven L. M. Schroeder, Anna Kroner, Federico Grillo, Stephen Francis, Paul Webb, Matthew Hand, Andrew Walters, Michael Hillman and Georg Held

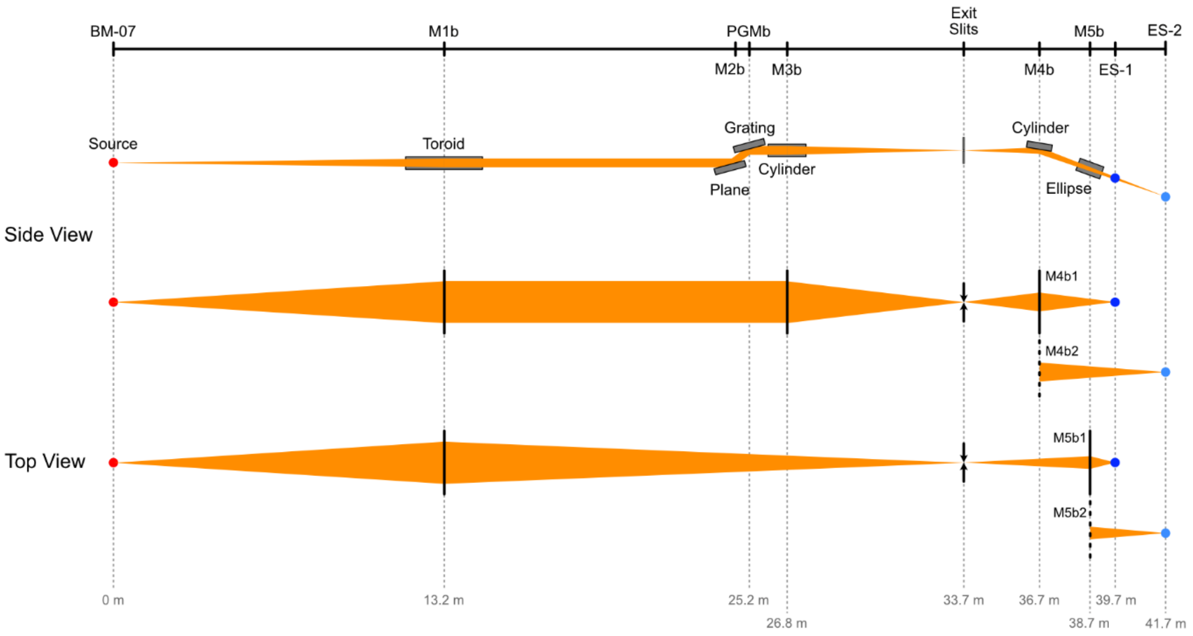

Figure S1 Optical layout of B07-B.

Table S1 Summary of the optical elements of B07-B.

Element	Surface Coating	Distance from Source (m)
M1b (Toroid)	Au	13.238
cPGM Mirror M2b (Plane)	Pt/Rh	24.748
cPGM Grating 400 l mm^{-1} (Laminar)	Ni	25.238
cPGM Grating 600 l mm^{-1} (Blaze)	Au	25.238
cPGM Grating 1000 l mm^{-1} (Laminar)	Ni	25.238
M3b (Cylinder)	Au	26.838
M4b1[2] (Cylinder)	Au [Au]	36.738
M5b1[2] (Ellipse)	Au [Pt]	38.738

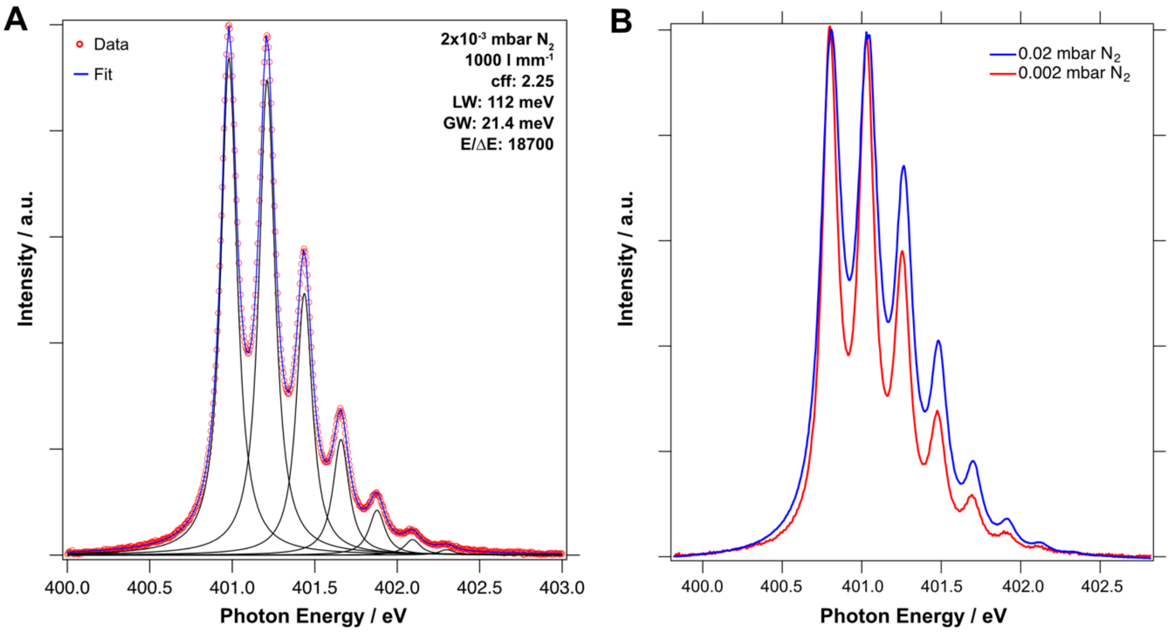

Figure S2 **A.** Nitrogen K edge XAS of gas phase N_2 showing a high-resolution scan of the 1s to π* transition with vibrational fine structure. Experimental data is shown as red circles, with the total fit as a blue line, and the 8 individual Voigt profiles as black lines. **B.** Nitrogen K edge XAS of N_2 as a function of pressure (red: 0.002 mbar, blue: 0.02 mbar)

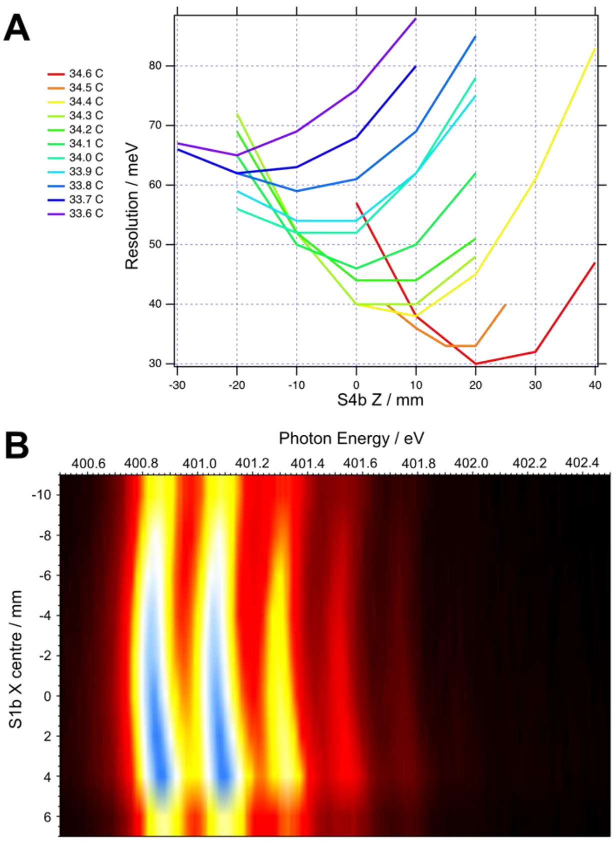

Figure S3 **A.** Plot of the beamline resolution at 400 eV photon energy as a function of exit slit translation along the beamline (S4b Z) at varying M1b chiller temperature setpoint. **B.** Nitrogen K edge XAS scans acquired as pencil slit scans (S1b X centre position) across M1b, demonstrating the shift in E_0 along the length of the mirror.

Figure S3B shows pencil scans of the Nitrogen K edge of N_2 gas using closed-down primary slits to show that different regions of the M1b surface have slightly different (~0.05 eV) apparent energy centres. This is likely due to variation in the collimation by M1b and is partially compensated by reducing the vertical acceptance of the PGM using a set of slits located immediately before the plane mirror (S2b, see Figure 1) and is ultimately the limiting factor in the photon energy resolution of the beamline.

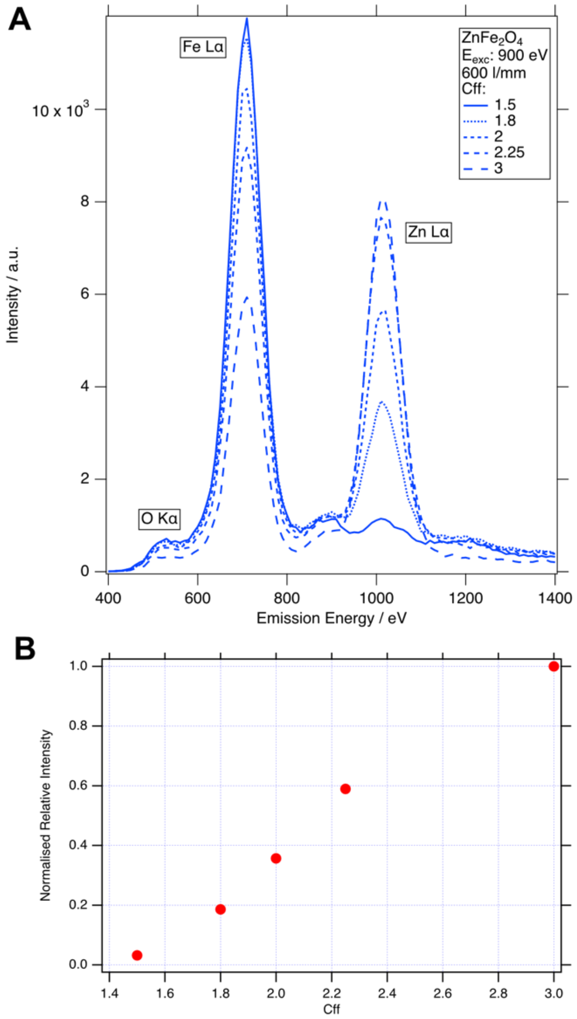

Figure S4 **A.** X-ray emission spectra of $ZnFe_2O_4$ as a function of the PGM cff. **B.** Intensity of the Zn L alpha emission line as a function of cff. (nominal hv = 900 eV)

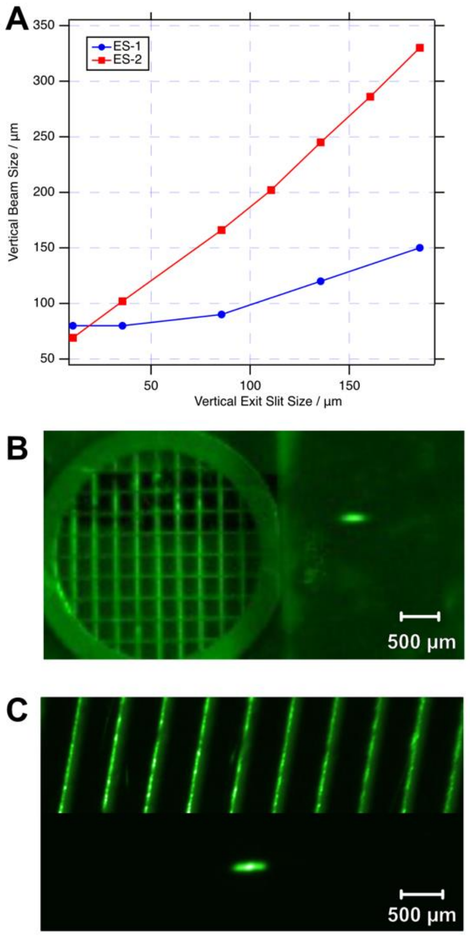

Figure S5 **A.** Vertical beam size at the two endstations as a function of vertical exit slit opening. (ES-1: blue, ES-2: red) **B.** Photograph of the normal-incident beam at ES-2, a TEM grid with a pitch of 250 µm is shown for size comparison. **C.** Photograph of the beam at ES-1, the lines at the top of the image are from a ruler with 500 µm spacing. This image is acquired at 60 degree incidence. (normal emission geometry for XPS)

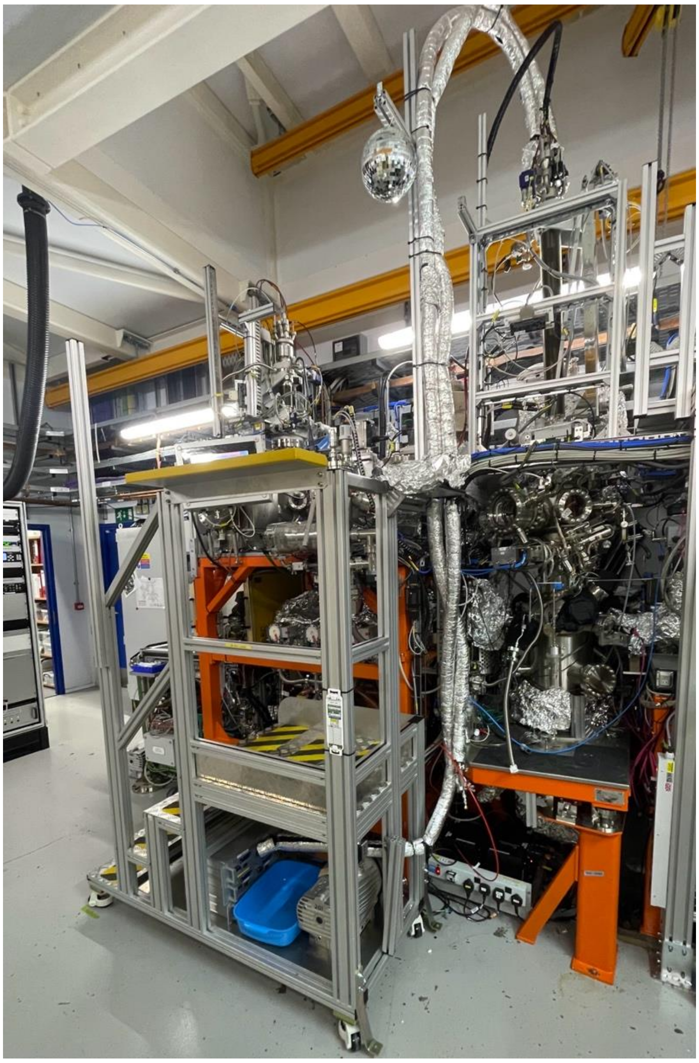

Figure S6 Photograph of ES-1.

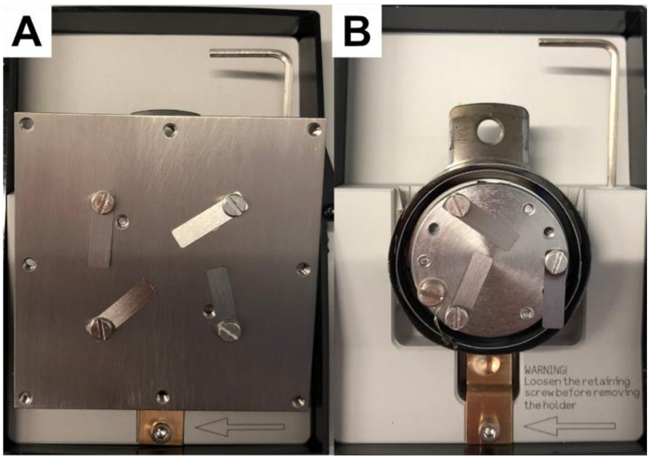

Figure S7 ES-1 sample plates. **A.** 50x50 mm^2 multi-sample plate. **B.** Single sample plate with resistive heater up to 1000 C.

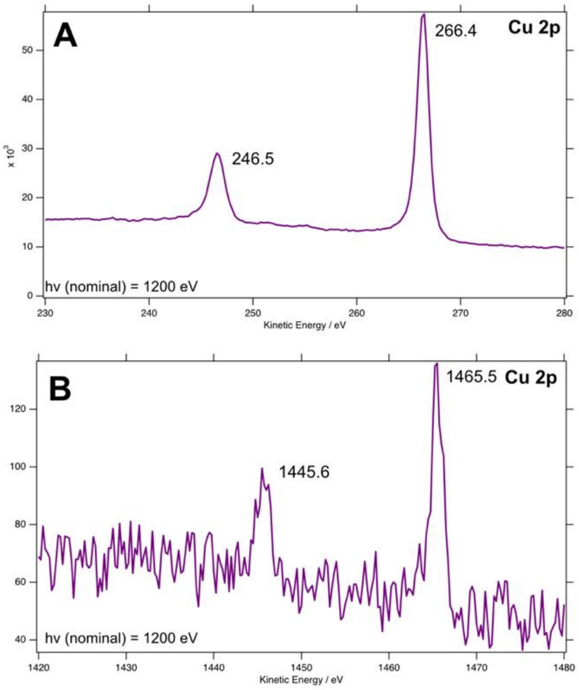

Figure S8 **A.** Cu 2p XPS of a copper foil. (nominal hv = 1200 eV) **B.** Cu 2p XPS of a copper foil with 2nd order light (~2400 eV).

After acquiring the spectrum of the region of interest (Figure S8A) a second spectrum is taken at a kinetic energy corresponding to double the nominal photon energy (Figure S8B). By measuring the difference in kinetic energies of the two spectra, the true photon energy can be calculated as shown below.

S1. 2nd Order Energy Calibration

(1) $h\nu = E_B + E_{K1} + \phi$

(2) $2 * h\nu = E_B + E_{K2} + \phi$

(2) – (1) $h\nu = E_{k2} - E_{K1}$

(2) – (1) $h\nu = 1465.5 - 266.4 = 1199.1\ eV$

$h\nu$: photon energy (eV)

E_B: binding energy (eV)

E_{K1}: kinetic energy with 1st order light (eV)

E_{K2}: kinetic energy with 2nd order light (eV)

ϕ: workfunction

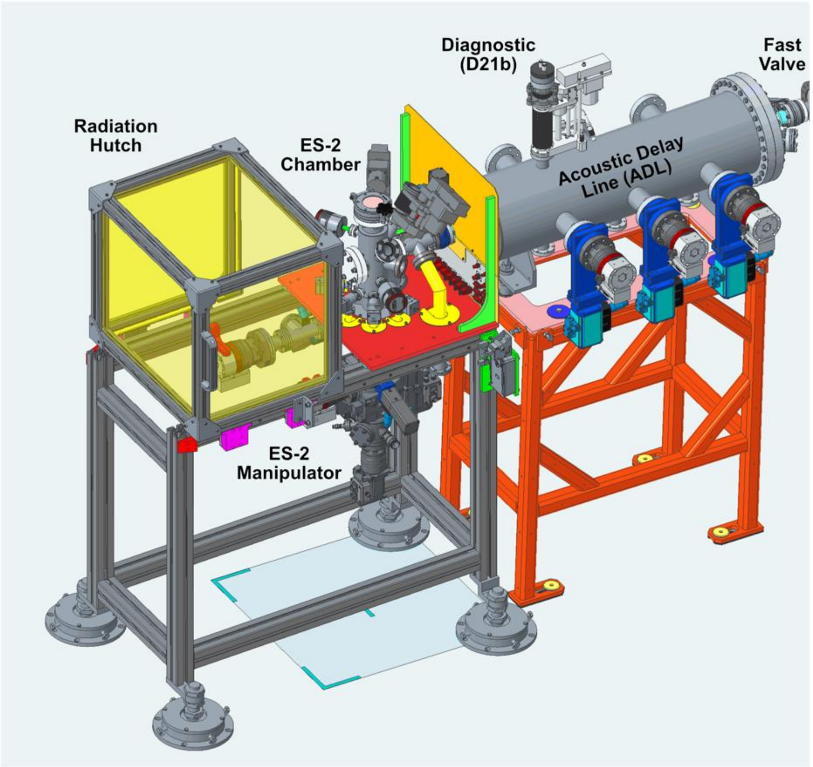

Figure S9 3D model of ES-2 and the acoustic delay line.

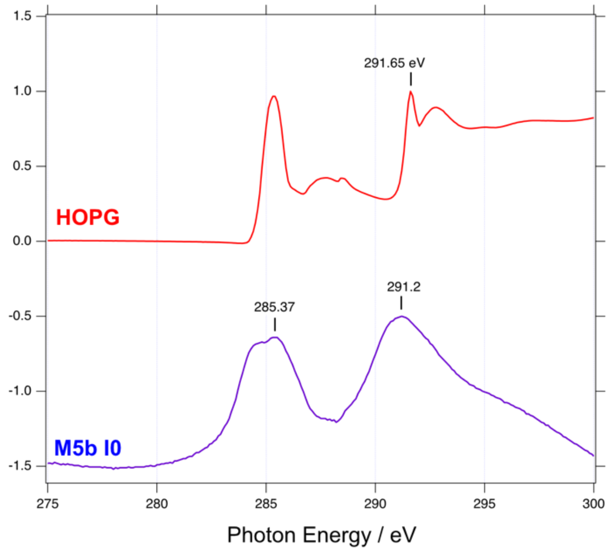

Figure S10 Carbon K edge energy calibration by comparison of HOPG with the beamline carbon structure as measured with the M5b I0.

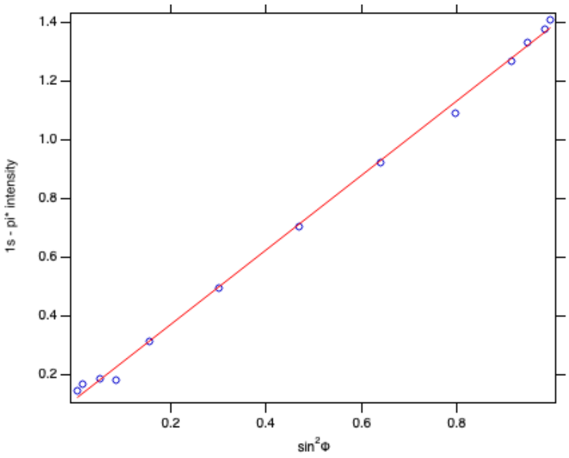

Figure S11 Plot of the HOPG pi* resonance intensity at 285.5 eV as a function of azimuthal angle, method as described in Watts et al. 2006.

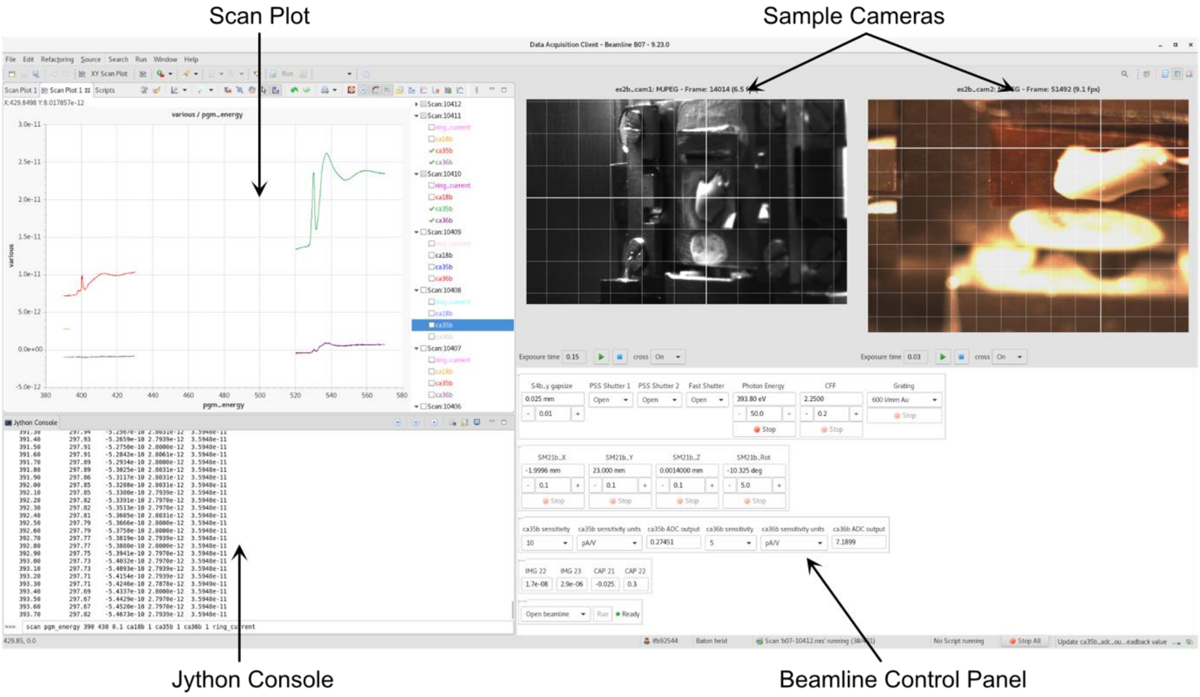

Figure S12 Screenshot of the ES-2 GDA perspective.
